# Is There an Association Between Dietary Antioxidant Levels and Sperm Parameters in Male Infertility?

**DOI:** 10.7759/cureus.44339

**Published:** 2023-08-29

**Authors:** Mehmet Taşkıran

**Affiliations:** 1 Urology, Özel Hatem Hastanesi, Gaziantep, TUR

**Keywords:** semen analysis, male factor infertility, total antioxidant capacity, astenospermia, oligospermia

## Abstract

Introduction: Oxidative stress is known as a mechanism underlying male infertility; it is defined as an imbalance between pro-oxidants and antioxidants leading to DNA damage, peroxidation of plasma membrane lipids, and protein oxidation. This study was conducted to investigate the relationship between total antioxidant capacity and sperm parameters in male infertility.

Methods: A total of 187 men with infertility (asthenospermia group (n=51), oligospermia group (n=40), and control group (n=96) were included in the current study. The following risk factors were recorded: age, sperm volume, sperm motility, hormone levels, and dietary antioxidant content.

Results: Demographic parameters and hormone levels of cases showed no statistically significant difference between groups (p > 0.05). Semen volume, motility, vitamin A, retinol, vitamin D, and vitamin C levels were statistically significantly lower in the asthenospermia and oligospermia groups (p < 0.05). According to the logistic regression model, lower vitamin A, retinol, vitamin D, and vitamin C levels were risk factors for poor sperm outcomes (p < 0.001).

Conclusıon: Male infertility with poor sperm outcomes should have an assessment of antioxidant capacity and nutritional specialization including food high in antioxidants could improve sperm parameters in asthenospermia and oligospermia and it could be used for therapeutic opportunities.

## Introduction

Infertility is defined as the inability to become pregnant after one year (or longer) of unprotected sexual intercourse. About one-third of infertility problems are due to the male partner. Semen analysis is the first stage of screening for male infertility in clinical practice [1]. Oxidative stress is known as a mechanism underlying male infertility; it is defined as an imbalance between pro-oxidants and antioxidants leading to DNA damage, peroxidation of plasma membrane lipids, and protein oxidation. Due to oxidative stress, reactive oxygen species (ROS) are formed, which impair sperm motility and lead to infertility, abnormal embryonic development, and possibly congenital fetal defects [2].

Total antioxidant capacity (TAC) is defined as the role of antioxidants required to reduce ROS, and its measurement provides useful information on the overall status of antioxidants, including those antioxidants that are not well measured or detected. In previous studies, dietary antioxidant levels were determined using a food intake frequency questionnaire [3].

The primary objective of this study was to investigate the correlation between dietary antioxidant levels and sperm motility, morphology, and sperm count in asthenoteratospermia and oligoasthenoteratospermia, in men with infertility.

## Materials and methods

This prospective study was conducted at the Department of Urology at Özel Hatem Hastanesi, Gaziantep, Türkiye, from January 2019 to January 2022. All patients gave written informed consent for the study, and the protocol was approved by the Gaziantep University Institutional Review Board (approval number: 2023/76).

A total of 187 men with infertility were included in this prospective study. They were divided into three groups: (i) asthenospermia group: n=51, (ii) oligospermia group: n=40, and (iii) control group: n=96. The groups were formed according to the spermıogram results. The following risk factors were recorded: age, sperm volume, sperm motility, hormone levels, and dietary antioxidant content.

Semen analysis was performed within one hour according to World Health Organization guidelines [4]. A hematoxylin-eosin (H&E) staining method was used to determine the percentage of normal sperm morphology. Samples were then classified as "normozoospermic", "asthenoteratospermic" (with motility < 50% and morphology < 14%), and "oligoasthenoteratospermic" (with sperm concentration < 20 million per ml, motility < 50%, and morphology < 14%). Sperm morphology was evaluated according to Kruger's criteria, according to which a morphology < 14% is considered abnormal [5].

At enrollment, all participants were informed about the study. Dietary antioxidant levels (DAL) were measured using the new 92-item antioxidant nutrient questionnaire developed by Satia et al. [3]. The results of the questionnaire were uploaded to the nutrient database program (BeBiS software program; Turkuvazsoft, Kayseri, Türkiye) developed for the evaluation of Turkish foods and convenience foods, and the validated nutrient contents were measured. Men who smoked and/or consumed alcohol, had leukocytospermia, pyospermia, diabetes mellitus, tuberculosis, and/or history of mumps, and those with a known male factor abnormality such as varicocele were excluded from the study.

Statistical analysis

Mean and standard deviation (SD) were calculated for continuous variables. The normality of variables was analyzed with the Mann-Whitney U test. The chi-square test and Student's t test were used to examine the associations between the categorical and continuous variables. The means of the three groups were analyzed using ANOVA and the Bonferroni post hoc test for multiple comparisons. The logistic regression method was used to determine the risk variables for the patients by including all variables in the model and calculating the odds ratios. All variables were included in the stepwise backward procedure. Two-tailed p-values were considered statistically significant at p <0.05 Statistical analyses were carried out by using SPSS for Windows, Version 15.0 (Released 2006; SPSS Inc., Chicago, United States).

## Results

During this study period, a total of 187 men with male infertility were evaluated. Table 1 demonstrates the demographic and clinical parameters of the patients. Demographic parameters and hormone values showed no statistically significant difference between the groups (p>0.05). Sperm volume, motility, vitamin A, retinol, vitamin D, and vitamin C levels were statistically significantly lower in the asthenospermia and oligospermia groups (p<0.05). According to the logistic regression model, lower vitamin A, retinol, vitamin D, and vitamin C levels were found to be significant risk factors for poor sperm results (P<0.001).

**Table 1:** Comparison of demographic and clinical characteristics between the groups TSH: thyroid stimulating hormone; FSH: follicle-stimulating hormone; LH: luteinizing hormone; PRL: prolactin

Variables	Asthenospermia group (n=51), mean±SD	Oligospermia group (n=40), mean±SD	Control group (n=96), mean±SD
Age	28.48 ± 6.12	27.85 ± 3.15	27.13±5.43
Volume (ml)	2.37 ± 0.26	1.67 ± 0.35	2.56±0.66
Progressive motility (%)	17.70±10.49	18.79 ± 11.76	22.27±9.43
Non-progressive motility (%)	13.9±9.41	12.28 ± 10.24	21.42±16.45
Immotile sperm (%)	42.45±10.53	34.09 ± 8.44	32.72±16.43
TSH (mU/mL)	3.33 ± 0.19	4.21 ± 0.24	3.26 ± 0.19
FSH (mIU/mL)	6.07 ± 0.64	4.11 ± 0.41	5.22±0.44
LH (mIU/mL	5.07 ± 0.25	5.33 ± 0.59	6.33 ± 0.39
PRL (ng/mL)	19.42 ± 0.62	17.60 ± 0.66	21.77 ± 0.62
Estradiol (pg/mL)	51.03 ± 0.29	62.42 ± 0.98	55.33 ± 0.54
Testosterone (nmol/L)	14.7 ± 3.2	17.9 ± 2.11	14.3 ± 3.8
Vitamin A (mg/L)	1.08±0.12	1.19±0.23	2.35±0.46
Retinol (mg/L)	0.54±0.26	0.64±0.28	2.52±0.60
Carotene (μg/ml)	1.64±0.34	1.24±0.74	1.44±1.00
Vitamin D ( ng/mL)	0.62±0.11	0.79±0.16	2.52±0.52
Vitamin E (μg/mL)	3.77±1.72	4.10±2.42	5.07±2.52
Vitamin B12 (pg/mL)	576.55±50.23	622.55±30.12	566.60±40.32
Vitamin C (mg/dl)	2.65±0.83	2.05±1.02	8.70±2.12

In subgroup analyses, 51 patients were asthenospermic and 40 patients were oligospermic. Table 2 summarizes the results of comparing the means of three groups by using ANOVA; this was followed by the Bonferroni test. There were no statistically significant differences between groups with regard to age, progressive and non-progressive motility, or hormone levels (including thyroid-stimulating hormone (TSH), follicle-stimulating hormone (FSH), luteinizing hormone (LH), prolactin, estradiol, and testosterone levels) (p > 0.05). Sperm volume, percent of immotile sperm,vitamin A, retinol, vitamin D, and vitamin C levels were statistically significantly different between groups (p < 0.05). Table 3 summarizes the outcomes of the logistic regression model performed in the subgroup. According to the model, vitamin A, retinol, vitamin D, and vitamin C levels were found to be significant risk factors between the asthenospermic and oligospermic groups (p < 0.05). For the asthenospermic group vs. control group, the odds ratio for vitamin A was 1.95, retinol 6.21, vitamin D 1.36, and vitamin C 1.86. Similarly, for the oligospermia group vs. control group, the odds ratio for those variables were 1.52, 5.47, 0.35, and 1.62, respectively.'

**Table 2:** Comparison of the means of three groups TSH: thyroid stimulating hormone; FSH: follicle-stimulating hormone; LH: luteinizing hormone; PRL: prolactin

Variables	Asthenospermia group (n=51), mean±SD	Oligospermia group (n=40), mean±SD	Control Group (n=96), mean±SD	F-value	p--value
Age	28.7±5.8	28.0±7.0	28.6±6.0	0.139	0.0871
Volume (ml)	2.37 ± 0.26	1.67 ± 0.35	2.56±0.66	1.193	0.001
Progressive motility (%)	17.70±10.49	18.79 ± 11.76	22.27±9.43	0.091	0.0914
Non-progressive motility (%)	13.9±9.41	12.28 ± 10.24	21.42±16.45	0.135	0.874
Immotile sperm (%)	42.45±10.53	34.09 ± 8.44	32.72±16.43	7.478	0.001
TSH (mU/mL)	3.33 ± 0.19	4.21 ± 0.24	3.26 ± 0.19	0,136	0.05
FSH (mIU/mL)	6.07 ± 0.64	4.11 ± 0.41	5.22±0.44	0,93	0.01
LH (mIU/mL	6.8±2.2	4.8±1.2	1.3 ± 0.5	5,47	0.05
PRL (ng/mL)	5.07 ± 0.25	5.33 ± 0.59	6.33 ± 0.39	1,24	0.08
Estradiol (pg/mL)	19.42 ± 0.62	17.60 ± 0.66	21.77 ± 0.62	2,35	0.07
Testosterone (nmol/L)	14.7 ± 3.2	17.9 ± 2.11	14.3 ± 3.8	4.6	0,08
Vitamin A (mg/L)	1.08±0.12	1.19±0.23	2.35±0.46	1.39	0.001
Retinol (mg/L)	0.54±0.26	0.64±0.28	2.52±0.60	0.15	0.009
Carotene (μg/ml)	1.64±0.34	1.24±0.74	1.44±1.00	0.135	0.874
Vitamin D ( ng/mL)	0.62±0.11	0.79±0.16	2.52±0.52	13.9	0.001
Vitamin E (μg/mL)	3.77±1.72	4.10±2.42	5.07±2.52	0.15	0.071
Vitamin B12 (pg/mL)	576.55±50.23	622.55±30.12	566.60±40.32	0.24	0.082
Vitamin C (mg/dl)	2.65±0.83	2.05±1.02	8.70±2.12	3.43	0.001

**Table 3:** Logistic regression analysis and outcomes

	Asthenospermia – Control group			Oligospermia – Control group		
Variables	Wald	OR (95% CI)	p-value	Wald	OR (95% CI)	p-value
Vitamin A (mg/L)	4.98	1.95 (0.70 – 5.55)	0.029	4.79	1.52 (0.59 – 3.98)	0.039
Retinol (mg/L)	16.33	6.21 (2.48 – 8.28)	0.001	14.23	5.47 (1.18 – 7.75)	0 .001
Vitamin D ( ng/mL)	3.21	1.36 (0.43 – 4.07)	0.001	2.82	0.35 (0.14 – 3.98 )	0.001
Vitamin C (mg/dl)	6.38	1.86 (0.66 – 3.57)	0.018	5.66	1.62 (0.49 – 4.12)	0.021

## Discussion

In the current study, 187 men with infertility were divided into three groups based on semen analysis: asthenospermia group; n=51, oligospermia group; n=40, control group; n=96. They completed an antioxidant diet questionnaire by Satia et al. [3]. This was a 92-item, self-administered questionnaire modeled after the semiquantitative food intake frequency questionnaire. There was a statistically significant difference between groups in age and vitamin A, retinol, vitamin D, vitamin E, and vitamin C contents (p < 0.05).

Previous studies have found an association between diet and poor spermiogram results. Moghadam et al. found that vitamin D deficiency in asthenozoospermic and healthy males can cause both apoptosis and necrosis and decrease progressive motility, total motility, and immobility [6]. We also found similar results in this study where vitamin D deficiency was a prognostic factor for poor spermiogram results.

In a randomized clinical trial, Kopets et al. demonstrated that a combination of several nutrients such as L-carnitine/L-acetyl carnitine, L-argi nin, glutathione, co-enzyme-Q, zinc, folic acid, cyanocobalamin, and selenium can improve sperm quality [7]. Several studies have also reported the importance of antioxidants in male infertility. Kobori et al. found that supplementation with vitamin C, vitamin E, and coenzyme Q10 resulted in significant improvement in sperm parameters [8]. Kodama et al. conducted a study and reported that the administration of vitamins E and C significantly decreased the hydroxyguanine content in sperm and resulted in increased sperm count [9].

Vitamin A, retinol, vitamin D, vitamin C, and vitamin E act as antioxidants in spermatogenesis. The oxidants released during spermatogenesis are neutralized by the antioxidants in the semen fluid. In our study, hormone levels (FSH, LH, testosterone) were similar in both groups and no statistically significant difference was found. Therefore, we can conclude that hormone levels do not affect antioxidant levels.

Antioxidants in semen include vitamin E, vitamin C, superoxide dismutase, glutathione, and thioredoxin. These antioxidants neutralize free radical activity and protect sperm from ROS that are already produced. Studies also indicate that high levels of reactive oxygen supplies caused by lower antioxidant levels also result in poor sperm parameters [10- 12]. Similar to these studies, in our study too, we found that men with lower antioxidants have poor sperm parameters.

Limitations of the current study include a low number of patients, a shorter follow-up period, and the lack of blood measurements of the antioxidant levels.

## Conclusions

The present study determines the role of oxidative stress and nonpharmacologic antioxidant therapies in male infertility. According to the current study, foods that contain more antioxidants may have a role in improving spermiogram results. Further studies with more participants are needed to establish new targets for novel treatments and provide potential benefits for male infertility patients.
